# The challenges of developing a contrast-based video game for treatment of amblyopia

**DOI:** 10.3389/fpsyg.2014.01210

**Published:** 2014-11-03

**Authors:** Zahra Hussain, Andrew T. Astle, Ben S. Webb, Paul V. McGraw

**Affiliations:** School of Psychology, University of NottinghamNottingham, UK

**Keywords:** anisometropia, binocular, contrast sensitivity, development, perceptual learning, strabismus, visual acuity

## Abstract

Perceptual learning of visual tasks is emerging as a promising treatment for amblyopia, a developmental disorder of vision characterized by poor monocular visual acuity. The tasks tested thus far span the gamut from basic psychophysical discriminations to visually complex video games. One end of the spectrum offers precise control over stimulus parameters, whilst the other delivers the benefits of motivation and reward that sustain practice over long periods. Here, we combined the advantages of both approaches by developing a video game that trains contrast sensitivity, which in psychophysical experiments, is associated with significant improvements in visual acuity in amblyopia. Target contrast was varied adaptively in the game to derive a contrast threshold for each session. We tested the game on 20 amblyopic subjects (10 children and 10 adults), who played at home using their amblyopic eye for an average of 37 sessions (approximately 11 h). Contrast thresholds from the game improved reliably for adults but not for children. However, logMAR acuity improved for both groups (mean = 1.3 lines; range = 0–3.6 lines). We present the rationale leading to the development of the game and describe the challenges of incorporating psychophysical methods into game-like settings.

## 1. Introduction

In this paper, we approach the challenge of creating a video game based on laboratory tasks shown to improve visual function in individuals with abnormal visual development (amblyopia). In amblyopia, monocular visual input is disrupted early in life due to misalignment of the ocular axes (strabismus), chronic blur in one eye (anisometropia), or a combination of the two. Visual acuity, contrast sensitivity and other visual judgments are reduced in the affected eye, and binocular function is degraded or absent (McKee et al., 2003; Levi et al., 2011). Amblyopia is a neural rather than optical disorder (Kiorpes and McKee, 1999; Barrett et al., 2004), and clinical treatment comprising occlusion of the non-amblyopic eye aims at strengthening the neural response to input from the amblyopic eye. This treatment is usually administered before 7 years of age, during the critical period of development when visual pathways in the brain are most malleable (Campos, 1995; Daw, 1998). Occlusion therapy can improve visual acuity in the amblyopic eye, but has issues of poor compliance (Holmes et al., 2003; Loudon et al., 2006; Wallace et al., 2013) and regression of improvements in up to a third of the cases (Hoyt, 2004). Therefore, supplementary treatments for amblyopia that surmount these issues continue to be of interest.

Practice of visual tasks can enhance visual function in amblyopic children and adults (Levi and Polat, 1996; Levi et al., 1997; Polat et al., 2004; Chen et al., 2008; Astle et al., 2011; To et al., 2011; Hussain et al., 2012b; Li et al., 2013). Such improvements in visual function after practice are classed under the phenomenon of “perceptual learning” and are attributed to residual plasticity in primary- and higher sensory cortices. In normally sighted individuals, perceptual learning is long-lasting and often specific to the trained task (for review see Sagi, 2011). In amblyopes, the improvements in addition to being long-lasting, generalize beyond the trained task to standard clinical measures of visual acuity and stereo acuity. The functional importance of perceptual learning for amblyopia has prompted a number of investigations into the task conditions that optimize improvements.

Visual acuity improves in amblyopia after practice on discrimination of single stimulus dimensions such as position, spatial frequency or contrast (Li and Levi, 2004; Polat et al., 2004; Astle et al., 2010, 2011), and after practice on commercial video games in which stimuli are far less constrained, but with which subjects are more likely to engage (Li et al., 2011; Jeon et al., 2012). Whilst psychophysical tasks help to isolate particular dimensions of improvement (e.g., resolution vs. contrast), video games provide more stimulating conditions that elicit the reward mechanisms associated with learning (Koepp et al., 1998; Suzuki et al., 2001; Dommett et al., 2005; Seitz et al., 2009; Rokem and Silver, 2010). From the psychophysical tasks tested to date, there is evidence that contrast sensitivity tasks are associated with the largest improvements on the trained task, and with the largest transfer of benefits to visual acuity (e.g., Polat et al., 2004; Chen et al., 2008; Astle et al., 2011, see Levi, 2012 for review). Practice need not be on contrast detection or discrimination *per se*, but on a visual judgment in which stimulus contrast is the dependent variable (e.g., Landolt C discrimination, Astle et al., 2011). There is also evidence that practice directed at the crowding problem in amblyopia (i.e., the inability to identify cluttered objects), improves visual acuity (Chung et al., 2012; Hussain et al., 2012b). In all above studies, the task was practiced monocularly with the amblyopic eye. A different approach involves practice with both eyes open, each viewing an independent stimulus (i.e., dichoptic viewing), with the aim of balancing the combination of visual input between the eyes (Knox et al., 2012; Li et al., 2013; Ooi et al., 2013). We chose monocular training to extend to more naturalistic settings work showing the success of this method in improving amblyopic acuity. Monocular methods also have the advantage of few hardware or software requirements, and minimal intervention or supervision of experts, and therefore are more practical to implement at home. With the above factors in mind, we developed a video game that incorporated the following features:

1. Dynamic pursuit of moving targets to maintain subject engagement
2. Adaptively varying target contrast
3. Multiple (crowded) targets and distractors
4. Spatially broadband targets at suprathreshold size
5. Monocular play with the amblyopic eye

To evaluate the effectiveness of this approach, we tested children and adults, who played the game at home for a minimum of 12 sessions. A subset of participants played the game for an extended number of sessions. We monitored performance on the game, and tested LogMAR acuity and stereoacuity before and after training.

## 2. Game concept

**Figure 1:**
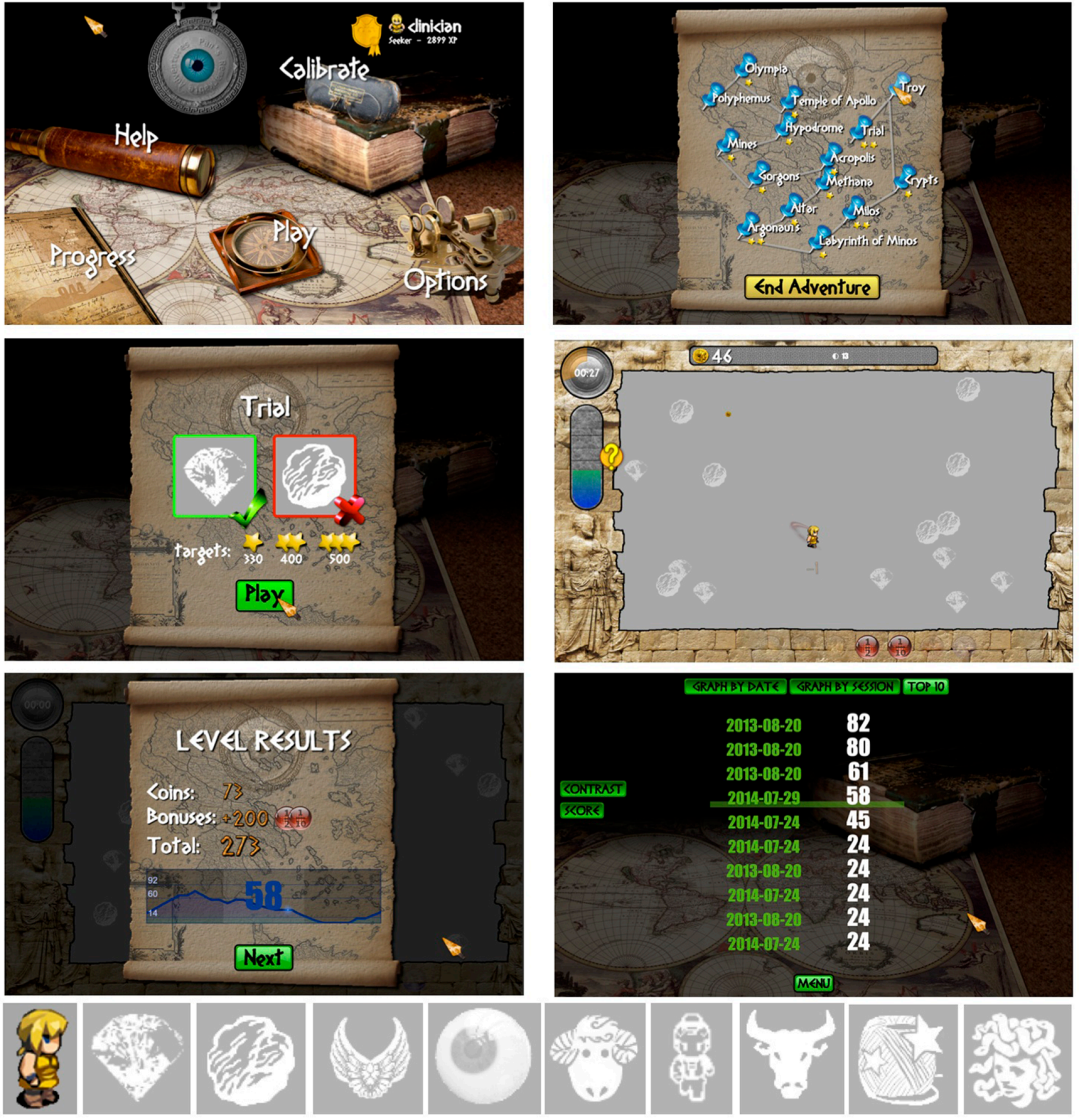
**Top left:** Main menu of game with links to all features. **Top right:** Each level in the game was shown as a destination on a map. **Middle left:** Screen showing the prize and enemy for the upcoming level. **Middle right:** Example of a game in progress. **Bottom left:** Summary of coin score and contrast sensitivity at the end of a level. **Bottom right:** Top 10 scores across multiple sessions. **Bottom:** Player's icon (Pan) and examples of prizes and enemies in different levels.

The game was called Pan's Remarkable Adventures, and can be accessed free of charge online at http://www.pangame.eu/beta. The game involves a central character, Pan, who travels across various destinations (or levels) in ancient Greece, collecting prizes, and avoiding enemies, with the goal of collecting as many coins as possible. We chose a collection-based game rather than a first-person shooter game to appeal to both genders, and to minimize violent content for younger age groups. Prizes and enemies varied across levels (Figure 1, bottom), as well as the configuration and motion of these objects. In each level, the player had 90 s to collect as many coins as possible, and to win stars that unlocked the next level. Progression across levels was visualized on a map showing each level as a destination (Figure 1). Players used a mouse to control Pan and pursue moving targets, and avoid colliding with moving enemies, both of whose contrast varied adaptively within each level. Players therefore had to discriminate and respond swiftly to targets and enemies of decreasing contrast. During an initial training phase, players were guided through a fixed sequence of levels on the map (Trial, Troy, Crypt, Milos, and Labyrinth of Minos). After three training runs, every training session always included the level Trial, which was used to track performance across sessions. The player was free to choose the remaining levels provided they had unlocked that level before. Free access to levels was designed to increase engagement with the game.

### 2.1. Reward

There were two types of reward. The first, a motivational reward, was a coin score directly linked to target and enemy collisions. Subjects won coins if they caught targets, and lost coins if they collided with enemies. When the player caught a target, high contrast gold coins materialized near that object and swept toward a score displayed at the top of the screen. More coins accumulated when targets and enemies were at low contrast, related to a second, contrast sensitivity score (see below). A progress bar on the left side of the screen increased as coins accumulated, and decreased when coins were lost. In addition to coins, auditory feedback and a number of bonus features (e.g., animated helpers, targets, and enemies slowing down) were included to maintain interest in the game.

The second type of reward was a performance-based score displaying contrast sensitivity on a scale of 1–100. This score was calculated from target contrast, which was adjusted continuously using a method described below. Whereas the coin score was derived from the absolute number of target and enemy collisions, the contrast score was based on the relative proportion of collisions, and provided a measure of target visibility. Subjects were told that when they were performing well, the targets would be difficult to see, and that they should try to improve both their coin score and their contrast sensitivity score. At the end of each 90 s level a graph was displayed to subjects showing changes in contrast sensitivity during that level.

Thus, good performance on the game was associated with a high coin score and with a high contrast sensitivity score, and the player's goal was to maximize both scores during each level. Bonus features affected only the coin score and not the contrast score. The top coin scores and contrast scores were saved for each subject and displayed on a “Top 10” page that subjects could access from the menu to view their past performance (Figure 1).

## 3. Materials and methods

### 3.1. Subjects

Ten amblyopic children (mean age = 9.3 years; *SD* = 2.4 years), and 10 amblyopic adults (mean age = 41 years; *SD* = 8.1 years) participated in the study. All subjects except one had at least a two-line (0.2 logMAR) difference in acuity between the amblyopic and the non-amblyopic (i.e., fellow) eye. One adult subject (*SD*) was bilaterally amblyopic and had equally poor acuity in both eyes. Six of ten children and six of ten adults had received patching treatment previously. Tables 1, 2 provide clinical details of the subjects. All subjects were informed of the purpose and procedure of the study, which was conducted under ethics approval from the School of Psychology at the University of Nottingham. All subjects provided a detailed ophthalmic history and were refracted by an optometrist before testing. LogMAR acuity (ETDRS) and Stereo acuity (TNO) were measured before and after training. Four adults obtained these measures from their local eye-care specialist before and after playing the game, and sent us the details through email. Two children were tested at Glasgow Caledonian University by a registered optometrist. Subjects who were not tested at the University of Nottingham are marked with an asterisk in Tables 1, 2.

**Table 1:** **Amblyopic children clinical details**.

**Initials**	**Age (yrs.)**	**Sex**	**Amb. eye**	**Type**	**Refractive error**	**Alignment (Diopters)**	**Patched**	**LogMAR PRE**	**LogMAR POST**
AB	8	M	L	Aniso+Micro	OD +1.50/−0.50 × 180			0.06	−0.10
					OS +3.50/−0.50 × 175	L Micro	Yes	**1.02**	**0.98**
BK	9	M	R	Strab	OD +7.00 DS	8Δ RSOT	Yes	**0.86**	**0.78**
					OS +5.50/−1.25 × 70			−0.10	−0.08
DP*	6	M	R	Strab	OD NA	18Δ RSOT	NA	**NA**	**NA**
					OS NA			NA	NA
LE	7	M	R	Strab	OD +7.75/−0.25 × 135	8Δ RSOT	Yes	**0.50**	**0.34**
					OS +8.00/−0.25 × 45			0.12	0.02
LG	9	M	L	Aniso	OD PL/−0.25 × 7			−0.10	−0.10
					OS +4.00/−4.50 × 170	–	No	**0.14**	**0.06**
MH	9	M	R	Strab	OD +1.00/−2.00 × 180	4Δ RSOT	Yes	**1.18**	**0.86**
					OS +2.00/−2.00 × 177			0.12	0.10
NJ	9	M	R	Aniso	OD +2.00/−0.50 × 125	R Micro	No	**0.20**	**0.08**
				+Micro	OS PL			−0.10	−0.08
OD*	8	F	L	Strab	OD +3.50/−0.50 × 90			0.02	0.02
					OS +3.50/−0.50 × 90	18Δ LSOT	NA	**0.36**	**0.36**
SR	10	M	R	Aniso	OD+4.50/−2.50 × 100	R Micro	Yes	**0.42**	**0.22**
				+Micro	OS +0.50/−0.75 × 85			0.00	0.00
WS	14	M	R	Aniso	OD +6.75/−1.75 × 165	R Micro	Yes	**1.36**	**1.32**
				+Micro	OS +0.25 DS			0.02	0.00

LogMAR acuity was measured using the ETDRS chart. Acuity of amblyopic eye shown in bold. Subjects marked with an asterisk were tested by an optometrist at Caledonian University.

**Table 2:** **Amblyopic adult clinical details**.

**Initials**	**Age (yrs.)**	**Sex**	**Amb. eye**	**Type**	**Refractive error**	**Alignment (Diopters)**	**Patched**	**LogMAR PRE**	**LogMAR POST**
AA*	42	M	L	Aniso	OD +1.00/−0.25 × 170			0.00	−0.08
					OS +3.00 DS	–	No	**0.22**	**0.18**
BM	36	F	R	Aniso	OD −0.75/−0.50 × 105	R Micro	No	**0.50**	**0.30**
				+Micro	OS −4.00/−0.50 × 120			0.06	0.04
IB	44	M	L	Strab + Aniso	OD +0.25/−0.25 × 110			−0.14	−0.18
					OS +4.50 DS	10 Δ LXOT	Yes	**0.52**	**0.36**
IT	42	M	L	Micro	OD +1.75/−0.75 × 90			−0.14	−0.08
					OS +3.25/−1.50 × 30	L Micro	Yes	**0.14**	**0.04**
JA*	24	F	L	Strab	OD +3.00 DS			−0.08	−0.08
					OS +4.50DS	12 Δ LSOT	Yes	**0.44**	**0.24**
JJ*	53	M	L	Micro	OD +4.00/−0.25 × 90			0.00	0.00
					OS +4.25/DS	Small LXOT	Yes	**0.30**	**0.24**
JL*	41	F	R	Aniso	OD +3.50/−0.25 × 45	–	Yes	**0.18**	**0.00**
					OS +0.75/−0.25 × 150			−0.08	−0.08
RC	46	M	R	Micro	OD +6.25/−1.75 × 10	R Micro	Yes	**0.42**	**0.22**
					OS +6.00/−2.50 × 175			−0.06	−0.06
RM	44	M	L	Aniso	OD −0.50/−0.50 × 120			−0.16	−0.14
				+Micro	OS +6.50/−6.25 × 85	L Micro	No	**0.16**	**0.12**
SD	37	F	B	Bilat.	OD +5.00/−0.50 × 100		Yes	**0.32**	**0.26**
					OS +3.00/−0.50 × 75			0.34	0.32

LogMAR acuity was measured using the ETDRS chart. Acuity of amblyopic eye shown in bold. Subjects marked with an asterisk were tested by their local eye-care specialist, and received the game instructions through email.

### 3.2. Apparatus and software

The game was written in HTML5 and JavaScript by Ilixa, a software development company (www.ilixa.com). Ilixa participated in discussions about the design of the game, created all stimuli and the game interface, and devised the algorithm for contrast adjustment based on adaptive staircase procedures (see Section 3.4.3). Subjects were given a demonstration of the game on an Apple G5 iMac computer, on Google Chrome. The monitor was a Trinitron Dell P1130 with a screen width of 40 cm and resolution of 1280 × 1024 pixels. A subset of subjects were also given the demonstration on their laptop computers, which they used to play the game at home. The training sessions were always on subjects' home computers, and included a variety of LCD displays ranging from large-screen television displays to smaller, laptop displays. No iOS devices (e.g., iPhone, iPad) were used. The game was always played with an external computer mouse.

### 3.3. Stimuli

The targets and enemies were broadband, suprathreshold size, gray-scale objects whose contrast varied adaptively against a uniform rectangular gray background (details on contrast follow). All other items in the display besides targets and enemies were colored objects shown at high contrast (see Figure 1, middle panels).

#### 3.3.1. Target size

Target size was set to 4.7% of the screen width in pixels, which ensured a constant number of targets and distractors on the screen across displays. On the laboratory display, target size at a viewing distance of 1 m subtended approximately 1° of visual angle. For the purposes of the game, it was important that target size was set above the acuity limit of the amblyopic eye, rather than being set to one size for all subjects. Hence, the viewing distance (and target size) was adjusted in the laboratory and in subjects' homes, to ensure that targets and distractors were discriminable. Subjects were given one meter as a rule of thumb for viewing distance, and were instructed to adjust this distance if needed to make the targets as discriminable as during the demonstration, and to maintain the same viewing distance across sessions.

At the above viewing distance and size, most subjects could discriminate targets from distractors during the demonstration and practice session. The experimenter ensured this was the case by monitoring performance during the practice run. A few subjects (e.g., AB, WS; Table 1), could not perform the task even after the viewing distance was decreased, therefore a two-alternative forced choice (2AFC) task that was included within the game was used to determine the size threshold for target-distractor discrimination for those subjects. A sample target and a sample distractor were shown at maximum contrast in adjacent positions on the screen for an unlimited duration, and the subject's task was to click on the target item. A three-down-one-up staircase was used to find the size discrimination threshold. For AB and WS, this procedure confirmed that targets could not be discriminated from distractors at the default size at the nearest viewing distance, and target size was increased to 1.25× and 1.8× the default in these subjects' game settings.

#### 3.3.2. Target-distractor configuration and motion

The motion, configuration and identity of targets and enemies on the screen varied from level to level. Targets were often diamond shaped objects and enemies were either spherical rock-like objects, or other objects that varied with the level (Figure 1). An example of the target and enemy for each level was shown at the start of each level. Target-distractor configurations ranged from being randomly intermingled on the screen (e.g., Trial), and forming mixed clusters (e.g., Olympia) to being placed in orderly, separated arrays (e.g., Milos). Target-distractor motion ranged from smooth translation in random directions across the screen, and bouncing movements, to situations in which targets eluded and/or enemies pursued the player.

### 3.4. Contrast

#### 3.4.1. Display calibration

**Figure 2:**
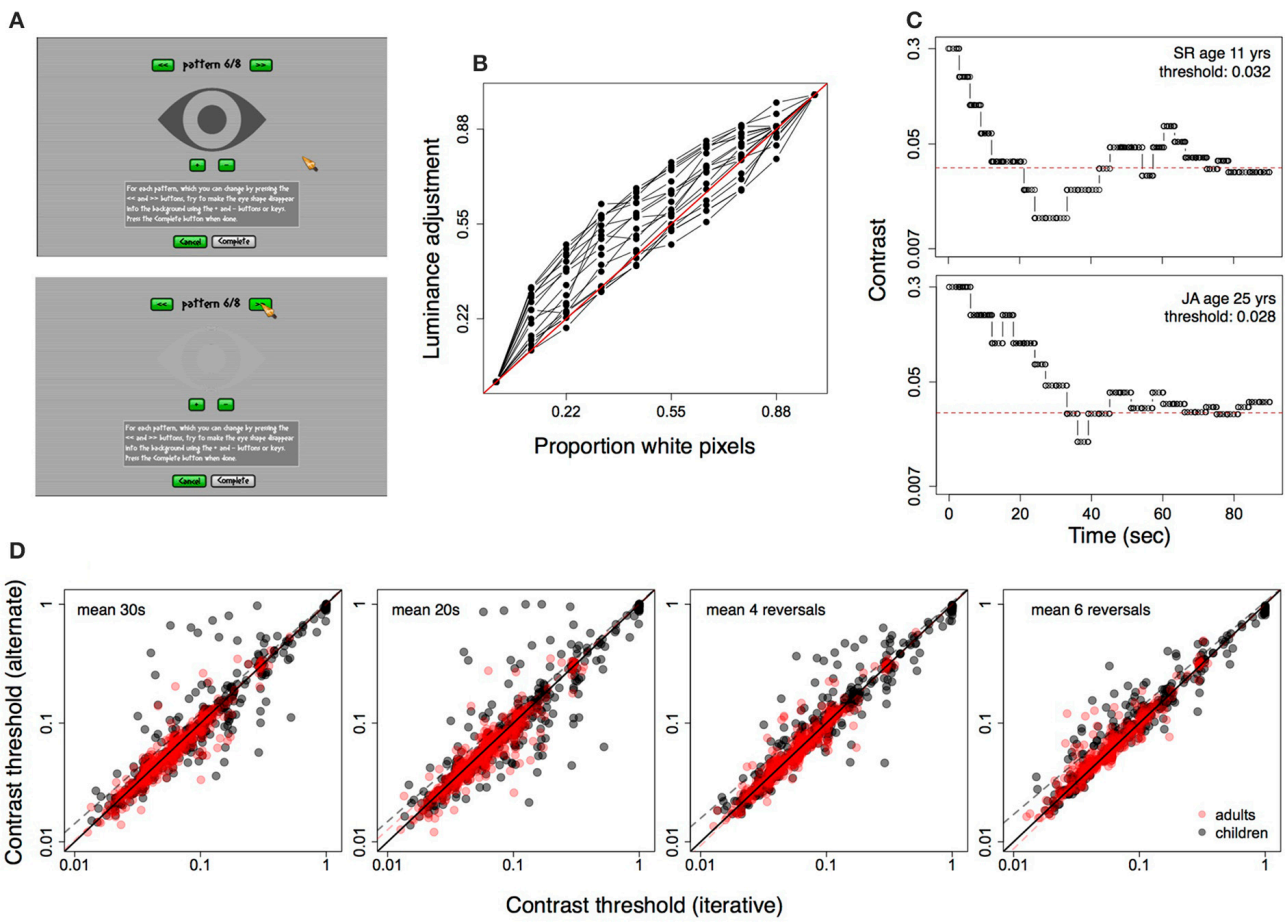
**(A)** Calibration screen showing central pattern matched to half-tone background using method of adjustment. Top: before adjustment, Bottom: adjusted pattern. **(B)** Calibration data for 18 displays, showing luminance adjustments (scaled from 0 to 1) against actual luminance increments (see text for details). **(C)** Contrast staircase from one level of the game for a child (top) and adult (bottom). **(D)** Correlation between contrast threshold calculated using iterative method (see text) and four other methods. From left to right: average contrast shown in final 30 s and final 20 s of the level, average of final four and final six contrast reversals.

Displays were calibrated using an observer-based procedure developed for LCD displays (Xiao et al., 2011). In this procedure, the observer matches a patch of uniform luminance to a half-tone background using the method of adjustment. Matches are repeated for eight luminance levels, and judgments of relative luminance are interpolated to correct for the display non-linearity (i.e., the opto-electronic transfer function, analogous to the gamma function in CRT displays). This procedure was completed through the “Calibration” option on the game home page. Subjects matched the brightness of a central eye-shaped pattern to that of the background by pressing on “+” or “−” buttons displayed on the screen, until the pattern blended into the background (Figure 2). Calibration was done in a lit room under the same conditions in which the game was played, and the entire procedure took about 5 min. Subjects were instructed to perform the calibration once prior to their first session, and to ensure that the display position and settings were unchanged across sessions. Figure 2 shows the adjustments obtained for eighteen subjects using this method. Gray values of the central image (rescaled from 0–255 to 0–1.00) selected by observers to match the background are plotted for each of eight background luminances. The background comprised black and white pixels (i.e., pixels set to zero and 255), and luminance was varied by increasing the proportion of white pixels (see Xiao et al., 2011). Thus, the curves are analogous to the inverse gamma function applied to correct for display non-linearities in CRT displays. The calibration settings were stored locally on subjects' computers and loaded each time the game was played.

#### 3.4.2. Contrast resolution and formula

Contrast resolution was increased beyond eight bits using an image dithering algorithm (Floyd and Steinberg, 1976), which is equivalent to adding imperceptible pixel noise to the target. Target pixels vary slightly around a mean value, rather than being set to a single value. These spatial fluctuations cannot be resolved by the eye, but change the effective contrast of the target against the background.

Contrast ranged between 0.00 and 1.00, and was defined as:

(1) $$c=\frac{L_{\text{target}}-L_{\text{min}}}{L_{\text{min}}}$$

where, *L*_target_ was the luminance of the target objects, and *L*_min_ was the minimum or background luminance, which was approximately 0.50. This formula for contrast is the same as Weber contrast.

#### 3.4.3. Contrast adjustment

Adjustment of contrast was modeled on standard staircase methods used in psychophysical experiments, and based on performance within successive 3-s time windows, each of which constituted a trial. Target contrast was adjusted using a probabilistic estimate of the subject's proportion of target collisions to total target and enemy collisions within the trial window. In a scenario where contrast must change adaptively based on events within a time window, it is more feasible to use a probabilistic estimate of performance than the actual number of collisions within each window. This is because the total number of target-distractor collisions within a short period may not be large enough to provide a precise measure of performance (e.g., zero hits, or 1/1 hit = 100% performance within 3 s), leading to large variations in contrast over time. Therefore, the method below was used to estimate the proportion of target collisions for the subject at a given contrast:

Let *p* be the number of targets collected, *e* be the number of enemies, and *n* the total number of collisions (*p* + *e*). Further, let *P*_*p*_ be the probability that any collision is a target, *p*. Two criteria, *T*_1_ and *T*_2_ were set, with *T*_1_ equal to 0.7 and *T*_2_ equal to 0.5. A contrast adjustment rule may be defined as:

– If *P*_*p*_ > *T*_1_, contrast decreases
– If *P*_*p*_ < *T*_2_, contrast increases

*P*_*p*_ cannot be estimated directly, therefore *p* and *e* were used to estimate the probability *P*_1_ (the probability that *P*_*p*_ is in range [*T*_1_, 1.00]) and *P*_2_ (the probability that *P*_*p*_ is in range [0.00, *T*_2_]).

These probabilities are estimated from the function:

(2) $$f_{\text{p,e}}(x)=x^{p}\;\cdot \;{\left(1-x\right)}^{e}\;\cdot \;\left(\begin{array}{c} p+e\\ p \end{array}\right)$$

where, *x* is the probability of a target collision.

From the above,

(3) $$P_{\text{1}}=\frac{\sum_{T1}^{1}f_{\text{p,e}}(x).dx}{\sum_{0}^{1}f_{\text{p,e}}(x).dx}$$

and

(4) $$P_{\text{2}}=\frac{\sum_{0}^{T2}f_{\text{p,e}}(x).dx}{\sum_{0}^{1}f_{\text{p,e}}(x).dx}$$

Now, if *P*_1_ is high and *P*_2_ is low, contrast decreases. Conversely, if *P*_1_ is low and *P*_2_ is high, contrast increases. This formulation was implemented discretely by setting *dx* to 0.001, and a stochastic variable was added to the contrast rule to smooth any sharp changes in contrast. Let *r* be a random number from a uniform distribution between 0 and 1.00:

– If *P*_1_ > 0.3 and *r* < *P*_1_ and *p* > 0, decrease contrast
– If *P*_2_ > 0.3 and *r* < 1 − *P*_2_ and *n* = 0 or *e* > 0, increase contrast
– Otherwise, keep contrast unchanged

Contrast was changed by multiplying or dividing by a multiplier (step size) to increase, or decrease contrast. The multiplier itself was adjusted based on performance in the previous three time windows (i.e., previous 12 s), with the starting (maximum) value set to 1.7, reaching a minimum of 1.12. Figure 2C shows examples of staircases constructed using the above method for one child and one adult subject during a single 90 s level.

#### 3.4.4. Contrast threshold calculation

The contrast threshold for each level was calculated using an iterative procedure from 30% of target contrasts nearest to the average target contrast over the 90 s duration. The threshold estimate included target contrasts from 27 s (though not necessarily contiguous) of the 90 s period. We compared this threshold measure with measures approximated from psychophysical procedures (i.e., average of the final four and six contrast reversals within the level) and with the average of all contrasts displayed in the final 20 and 30 s of the level. Figure 2D shows the above four measures of threshold plotted against the iterative measure for all subjects, from all sessions, on one level of the game. Thresholds from the iterative method were strongly correlated with the other measures (Pearsons's *r* > 0.80, *p* < 0.0001 for all tests).

### 3.5. Procedure

Subjects were refracted and a full ophthalmic history obtained. Subjects were fitted with their best optical correction for the demonstration and an initial practice run on the game, and were instructed to use their best correction when they played the game at home. The game was always played monocularly, with a patch over the fellow eye. Demonstration of the game included subject registration (setting up an ID and password that was used each time the subject played the game), use of the game menu, display calibration, ensuring that target size was set above the acuity limit of the amblyopic eye, and a practice run on the game. During the practice run, the experimenter confirmed that target contrast decreased adaptively from its starting point, indicating that subjects understood how to play the game. Detailed instructions were given on how to set up the game at home. Subjects were told to download the browser Chrome if it did not already exist on their computers, to set their computer monitors to a fixed viewing distance (1 m was suggested, but also see stimulus size section above) for all sessions, and to calibrate their display using the demonstrated method prior to the first session. For children, parents set up the game and confirmed that the instructions were being followed. For subjects who did not attend a demonstration at the University of Nottingham or Glasgow Caledonian University (i.e., four adults), detailed instructions of all steps were provided in a separate document sent through email. Subjects were instructed to play the game every day, for at least 25 min a day. Visual acuity was re-measured after at least 2 weeks of training. A subset of subjects (six children and four adults) continued to play the game after the first acuity re-test, and returned for a second re-test some weeks later.

### 3.6. Data storage

Each subject had a unique ID, which they used each time they played the game. Their data from each session were stored on a server, which could be accessed by the experimenters at the University of Nottingham. The data for each subject included date of session, duration of session, number of levels played at each session, names of the levels played and the contrast threshold for each level. Hardware and software details were also stored, including the OS, browser name and version, window size (in pixels), and monitor refresh rate. Thus, the experimenter was able to keep track of whether subjects were playing the game regularly, and for a minimum duration each session.

### 3.7. Contrast thresholds

**Figure 3:**
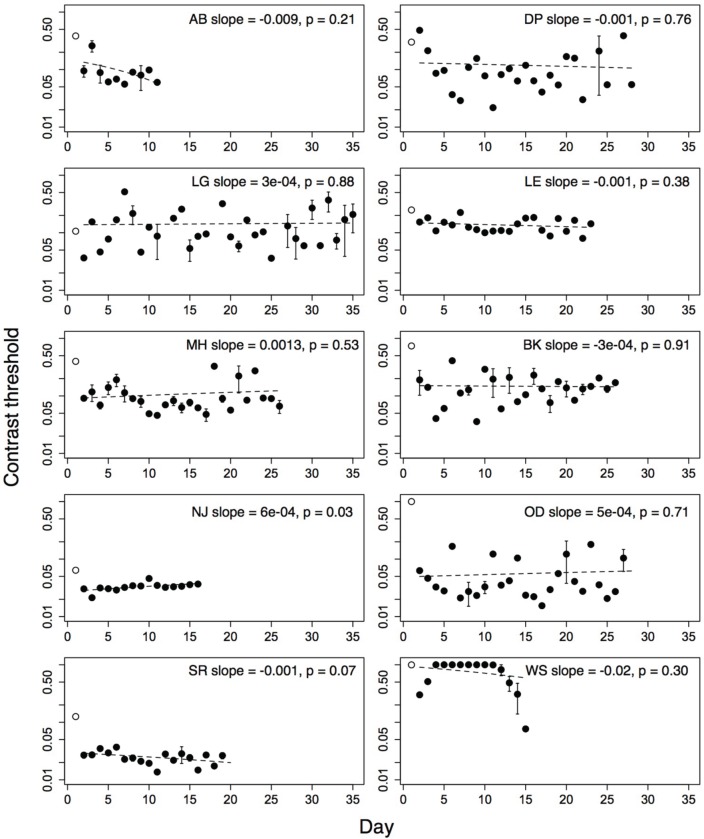
**Performance of 10 amblyopic children on level Trial over multiple days**. Open symbol shows performance on first demonstration session. Error bars show standard error of the mean (s.e.m.), where the subjects played more than one run. Dashed line shows regression fit of threshold on day. Open symbol not included in fit. Subject initials, slope of the regression fit and associated *p*-value given in each plot.

**Figure 4:**
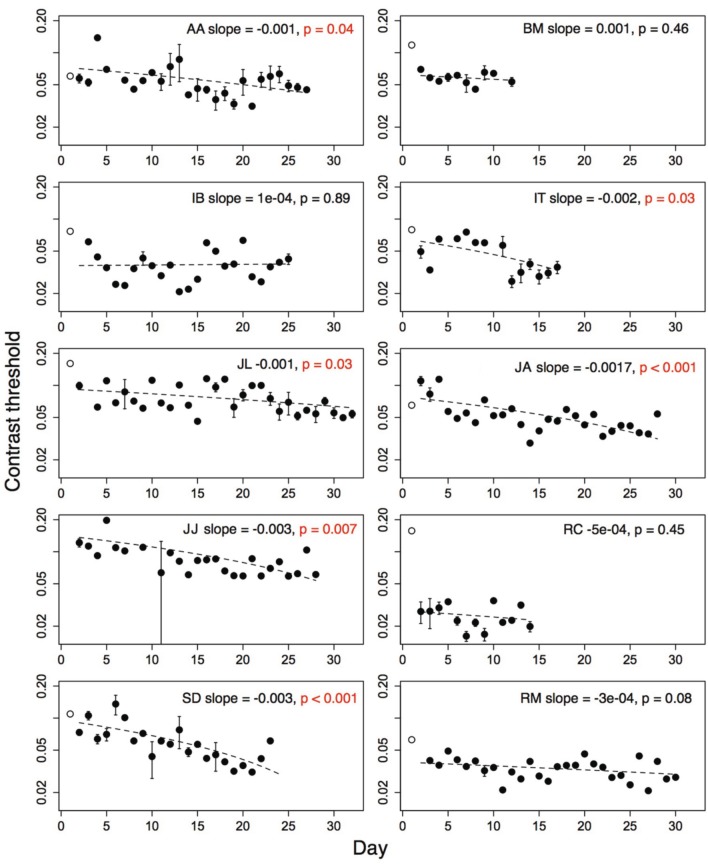
**Performance of ten amblyopic adults on level Trial over multiple days**. Open symbol shows performance on first demonstration session. Significant improvement indicated by *p*-values in red. Error bars show standard error of the mean (s.e.m.), where the subjects played more than one run. Dashed line shows regression fit of threshold on day. Open symbol not included in fit. Subject initials, slope of the regression fit and associated *p*-value shown.

Contrast thresholds from the Trial level, which was obligatory on each run, were used to measure improvement on the game. Contrast thresholds were always measured for the amblyopic eye, with a patch over the fellow eye. For each subject, outlying threshold values (i.e., thresholds more than two standard deviations greater than or less than the mean threshold over all sessions of that subject), were removed from the analyses. Five percent of the children's thresholds and four percent of the adults' thresholds were removed using this criterion. The number of training days between the pre- and post-training acuity tests ranged from twelve to thirty-two (mean = 23.4; *SD* = 7.21). Thresholds from the very first day (open symbols, Figures 3, 4), were discarded from the analyses as they were measured during the initial demonstration, on a different display than the display used for the remaining sessions. The second day's threshold was considered as the initial threshold value. We measured the amount of improvement as the difference between thresholds on the initial and final training day (i.e., initial threshold minus final threshold). We also calculated learning slopes from linear regression of threshold against day for each subject. According to this measure, improvement is associated with a negative slope significantly different from zero. Improvement on perceptual tasks has also been suggested to increase as a power- or exponential function of the amount of practice (Dosher and Lu, 2007). We compared the fit of a linear model and a decaying exponential model of threshold against session number for each subject using the Akaike Information Criterion (AIC). This criterion produced better exponential fits than linear fits for three of ten adult subjects, and one of ten children (child: DP; adults: BM, IB, JO). For the analyses that follow, we report the linear fits for all subjects.

## 4. Results

### 4.1. Contrast thresholds

**Figure 5:**
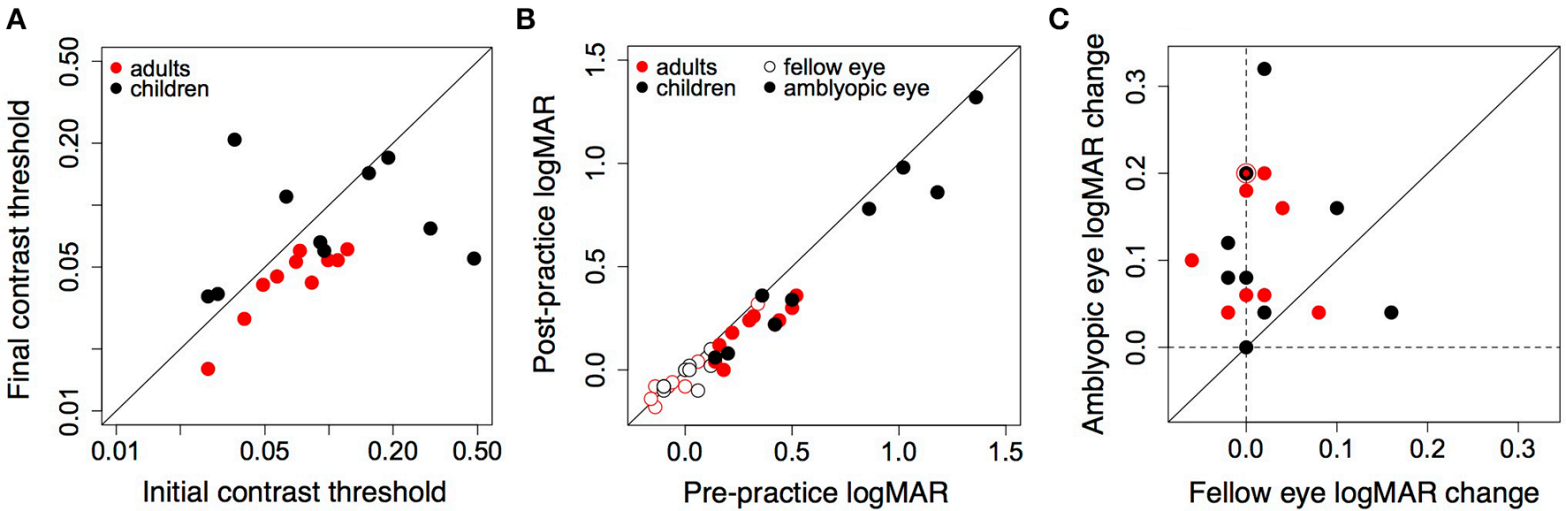
**(A)** Initial and final contrast thresholds measured on the game for adults and children. Points below the diagonal show improvement. **(B)** Pre- and post-training LogMAR acuity of the amblyopic eye (closed symbols) and fellow eye (open symbols) of 9 amblyopic children and 10 amblyopic adults. Points below the diagonal show improvement. **(C)** Improvement of the amblyopic eye against improvement in the fellow eye. Dashed vertical and horizontal lines show zero change. Points above the diagonal show greater improvement in the amblyopic eye than fellow eye. The black and red symbol shows data for two adults and one child. Sixteen of nineteen points lie above the unity line.

Performance of amblyopic children and adults on all training days is shown in Figures 3, 4. Figure 5A summarizes the threshold data. The average contrast threshold of children changed from 0.15 (*SE* = 0.05) on the initial day to 0.09 (*SE* = 0.02) on the final day. This change in threshold between days was not significant [*t*_(9)_ = 1.021, *p* = 0.33]. As seen in Figure 3, there was substantial variability in children's performance across days, and the learning slope did not differ from zero for any individual child. Contrast thresholds decreased from the initial to final measurement for the adults [Figures 4, 5A, from 0.07 (*SE* = 0.01) to 0.045 (*SE* = 0.005)]. This change in threshold was significant [*t*_(9)_ = 4.20, *p* = 0.002]. Figure 4 shows that the improvement in adults was more reliable than in children, with the learning slope for six of ten adults significantly different than zero. Overall these data suggest that for the adults, but not for children, the contrast threshold was a reliable measure of performance on the game. Potential explanations for this difference are discussed later.

### 4.2. LogMAR acuity

LogMAR acuity of the amblyopic eye and the fellow eye of the nineteen subjects is shown in Figure 5B (acuity data were not available for one child participant who was tested outside Nottingham). Acuity of the amblyopic eye improved by 0.12 logMAR both for children [*t*_(8)_ = 3.51, = 0.008], and adults [*t*_(9)_ = 5.57, *p* = 0.00034]. Acuity of the fellow eye did not change significantly after training either for children [*t*_(8)_ = 1.43, *p* = 0.19] or adults [*t*_(9)_ = 0.6882, *p* = 0.51]. Figure 5C plots the improvement in acuity of the amblyopic eye against the change in acuity of the fellow eye for all subjects. Improvement in the amblyopic eye outweighed improvement in the fellow eye for all but three subjects, and improvements in the acuity of the amblyopic eye were not correlated with the change in acuity of the fellow eye. Therefore, subjects were not merely improving at reading the letter acuity chart. For four mild amblyopes (children: LG and NJ, initial acuity: 0.14 and 0.20, Table 1; adults: IT and JL, initial acuity: 0.14 and 0.18, Table 2), the difference in visual acuity between the eyes was reduced to less than two lines, rendering them no longer amblyopic according to the criterion of the study. Table 3 gives the correlation coefficient (Pearson's *r*), and *p*-values for correlations between absolute amount of logMAR improvement and threshold change, initial logMAR acuity, logMAR improvement in the fellow eye, number of sessions, session duration, total hours played and the age of the participant. Improvements in logMAR acuity were not correlated with most measures, except for a marginally significant positive correlation between initial logMAR acuity and logMAR improvement of the adults (suggesting that poorer starting acuity was associated with larger improvement), and marginally significant associations between improvement and session duration, and improvement and total hours played in children (see below).

**Table 3:** **Correlation between change in LogMAR acuity and other variables**.

	**Children**	**Adults**
Threshold improvement	−0.05 (0.90)	0.26 (0.47)
Initial LogMAR acuity (amblyopic eye)	0.12 (0.76)	**0.63 (0.05)**
LogMAR improvement in fellow eye	−0.09 (0.81)	−0.08 (0.82)
Number of training sessions	0.11 (0.78)	−0.40 (0.26)
Median minutes per session	**0.61 (0.08)**	−0.15 (0.67)
Total hours played	0.53 (0.15)	−0.37 (0.28)
Total hours after extended practice	**0.66 (0.05)**	−0.04 (0.91)
Age	−0.05 (0.89)	−0.39 (0.26)

Correlation coefficient r, and p-value shown. Acuity tested multiple times for extended practice subjects. Values in parentheses are *p*-values.

### 4.3. Extended training, session duration, and total amount of practice

**Figure 6:**
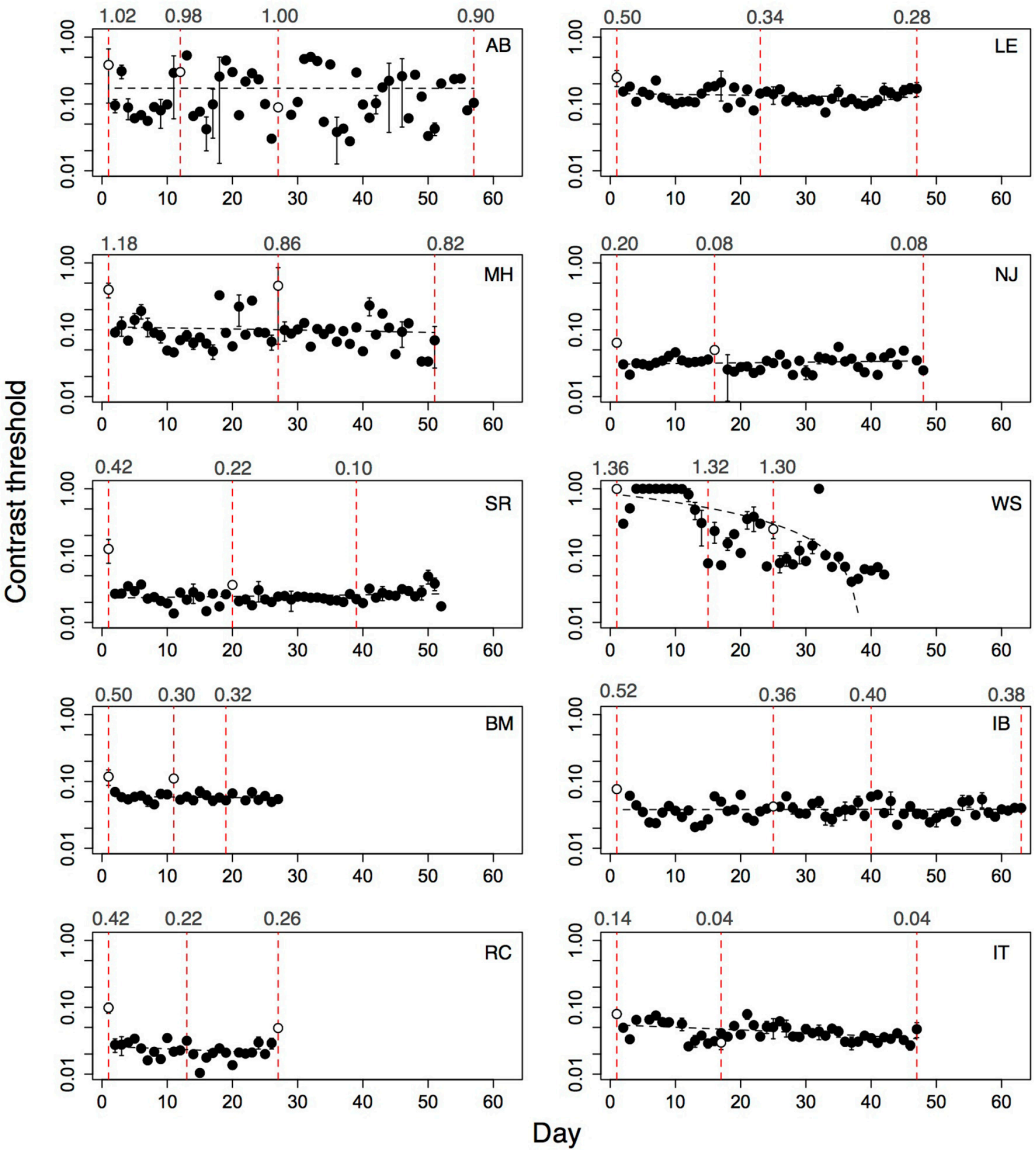
**Performance of ten amblyopic subjects who played for an extended number of days, and whose acuity was re-tested more than once**. Top six panels show children, bottom four panels show adults. Closed symbols represent thresholds from subjects' home computers. Dashed black line: regression fit of threshold over day. Open symbols show thresholds measured in the laboratory and are not included in the fit. Vertical red dotted lines: days on which logMAR acuity was tested. Numbers at top of dashed lines: logMAR acuity. See text for analyses.

A subset of subjects (six children and four adults) played the game for additional days and returned for a second acuity re-test. Performance of these subjects on the game is shown in Figure 6. For these subjects, contrast thresholds did not change significantly between the logMAR pre-test and the first post-test [mean difference = 0.02; *t*_(8)_ = 0.82, *p* = 0.44], or between the first post-test and the second post-test [mean difference = 0.009; *t*_(8)_ = 0.52, *p* = 0.61]. For all subjects except subject WS, learning slopes did not differ from zero. Between the pre-test and first post-test, logMAR acuity of these subjects improved by 1.5 lines [mean = 0.15; *t*_(8)_ = 4.6904, *p* = 0.002]. After additional practice, logMAR acuity improved further by a small amount for children [mean = 0.05, *t*_(5)_ = 3.02, *p* = 0.02; post-test1 minus post-test2], but did not change for adults [mean = −0.02, *t*_(5)_ = 2.45, *p* = 0.09]. The total improvement in thresholds from the initial to the final session of these subjects was not correlated with their improvement in logMAR acuity from pre-test to the second post-test [*r* = 0.46; *t*_(7)_ = 1.40, *p* = 0.20]. These data suggest that extended play of the game produced slight additional improvements in logMAR acuity only for the children, and that the improvements in logMAR acuity of both groups were maintained over the testing period.

**Figure 7:**
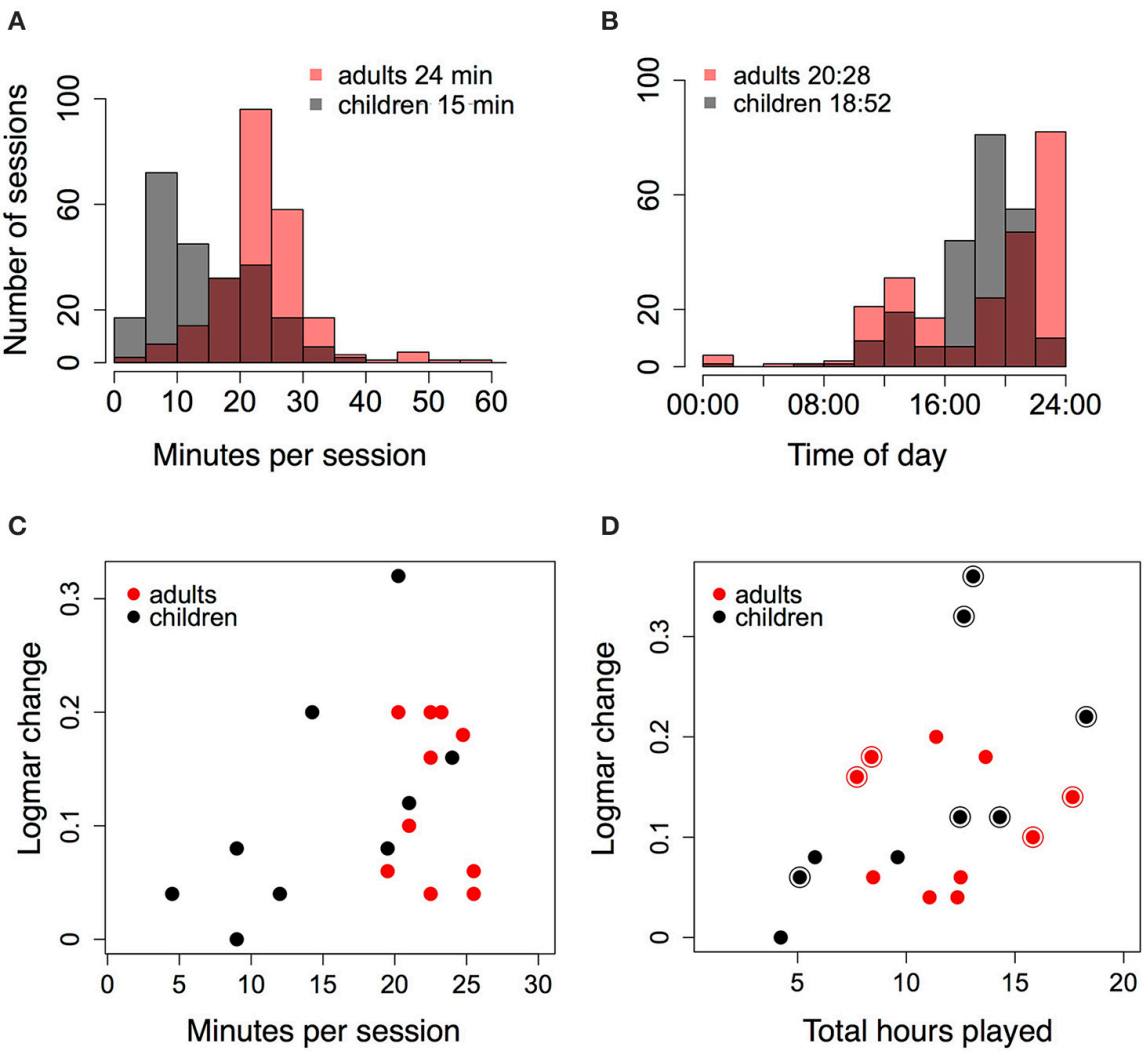
**(A)** Histogram showing the amount of time played per session by children and adults. Legend shows mean number of minutes played each session. **(B)** Histogram showing time of day at which children and adults played the game. Legend gives median time. **(C)** Change in logMAR acuity for each subject (at first acuity post-test) against median minutes per session. **(D)** Change in logMAR acuity for each subject against total hours played over all sessions. Large symbols show subjects who played for an extended duration, and whose acuity was re-tested more than once.

Did the duration of each session affect the amount of improvement? Figures 7A,B show histograms of session duration (in minutes), and and the time of day at which subjects played the game, for all sessions of all subjects. Adults played the game for approximately 24 min on average per session, which was significantly longer than children, who played for an average of 15 min per session [*t*_(462.249)_ = 12.05, *p* < 0.0001]. Therefore, adults, but not children, complied with the suggested duration of practice on the game. Both groups played the game during the latter part of the day, and adults played later than children (median hour: 20:28 vs. 18:52; Wilcoxon rank sum test; *W*= 32311.5, *p* = 0.00026). Figure 7C shows the relationship between median session duration for each subject and their logMAR acuity improvement. The correlation between improvement in logMAR acuity and duration was marginally significant for children [*r* = 0.61, *t*_(7)_ = 2.01, *p* = 0.08], but not adults [*r* = −0.15, *t*_(8)_ = 0.44, *p* = 0.67]. Note that median duration for adults always exceeded 15 min, and that the range of durations was fairly narrow (Figure 7C).

We examined whether larger amounts of practice, as measured by the total number of hours played by each subject, were associated with larger improvements in logMAR acuity. Figure 7D shows logMAR improvement for all subjects against the total number of hours played. These data include the extra hours played by subjects who trained for additional sessions after their first acuity post-test, and whose acuity was tested more than once (final acuity and number of hours shown for every subject). Figure 7D suggests that larger improvements in logMAR acuity were associated with more hours played; this correlation was marginally significant across all subjects [*r* = 0.41, *t*_(17)_ = 1.87, *p* = 0.08]. When considered for each group separately, the correlation between hours played and logMAR improvement was not significant for adults [*r* = −0.04, *t*_(8)_ = −0.11, *p* = 0.91], and marginally significant for children [*r* = 0.66, *t*_(7)_ = 2.34, *p* = 0.05]. Subjects whose acuity was re-tested more than once are indicated in Figure 7D (large symbols), and span the range of logMAR improvements, suggesting that this relationship was not based on acuity re-tests alone. Furthermore, a similar pattern was evident (but not significant) when the number of hours between pre-test and first post-test were considered (see Table 3, Total hours played, Total hours after extended practice), suggesting that the total amount of practice did matter.

Overall, the data suggest that for children, clinically significant improvements in logMAR acuity may depend on a minimum amount of practice per session, and on the total amount of practice across all sessions. The number of sessions was not a good predictor of acuity improvement in children because it was uncorrelated with session duration and total hours played. The relationship between acuity improvement and session duration was less clear in adults, possibly because of the narrow range of durations across subjects. Large improvements in adults also may require far greater amounts of practice than are needed for children, greater than the maximum amount measured in this study. Additionally, the improvement in adults may be limited by other factors than the amount of practice, such as the depth of amblyopia and the properties of the training task. More data including a broader range (and manipulation) of session durations and total amounts of practice are needed to clarify this issue.

### 4.4. Stereoacuity

Stereoacuity was measured using the TNO test for stereoscopic vision before and after training. Stereoacuity improved from 60 to 30 s of arc for one child, from 120 to 60 s of arc for two children, and from more than 30 min of arc (No Stereo) to 30 min of arc (Gross Stereo) for two children. There was no change in stereoacuity in the other five children. Stereoacuity changed from No Stereo to 480 s of arc for one adult, from No Stereo to Gross Stereo for another adult, and did not change in the other adults. We grouped subjects according to whether their stereo acuity changed (Group 1, *N* = 7) and did not change (Group 2, *N* = 11) after game play. There was no difference between these groups in amount of change in logMAR acuity [*t*_(14.98)_ = 0.43, *p* = 0.67], median minutes per session [*t*_(15.496)_ = 0.40, *p* = 0.69], hours played on the game [*t*_(15.37)_ = 0.49, *p* = 0.62], and number of sessions played [*t*_(10.1)_ = 0.1.27, *p* = 0.23]. These results suggest that improvements in stereo acuity could not be predicted from changes in letter acuity, or the amount of practice.

### 4.5. Level difficulty and preference

**Figure 8:**
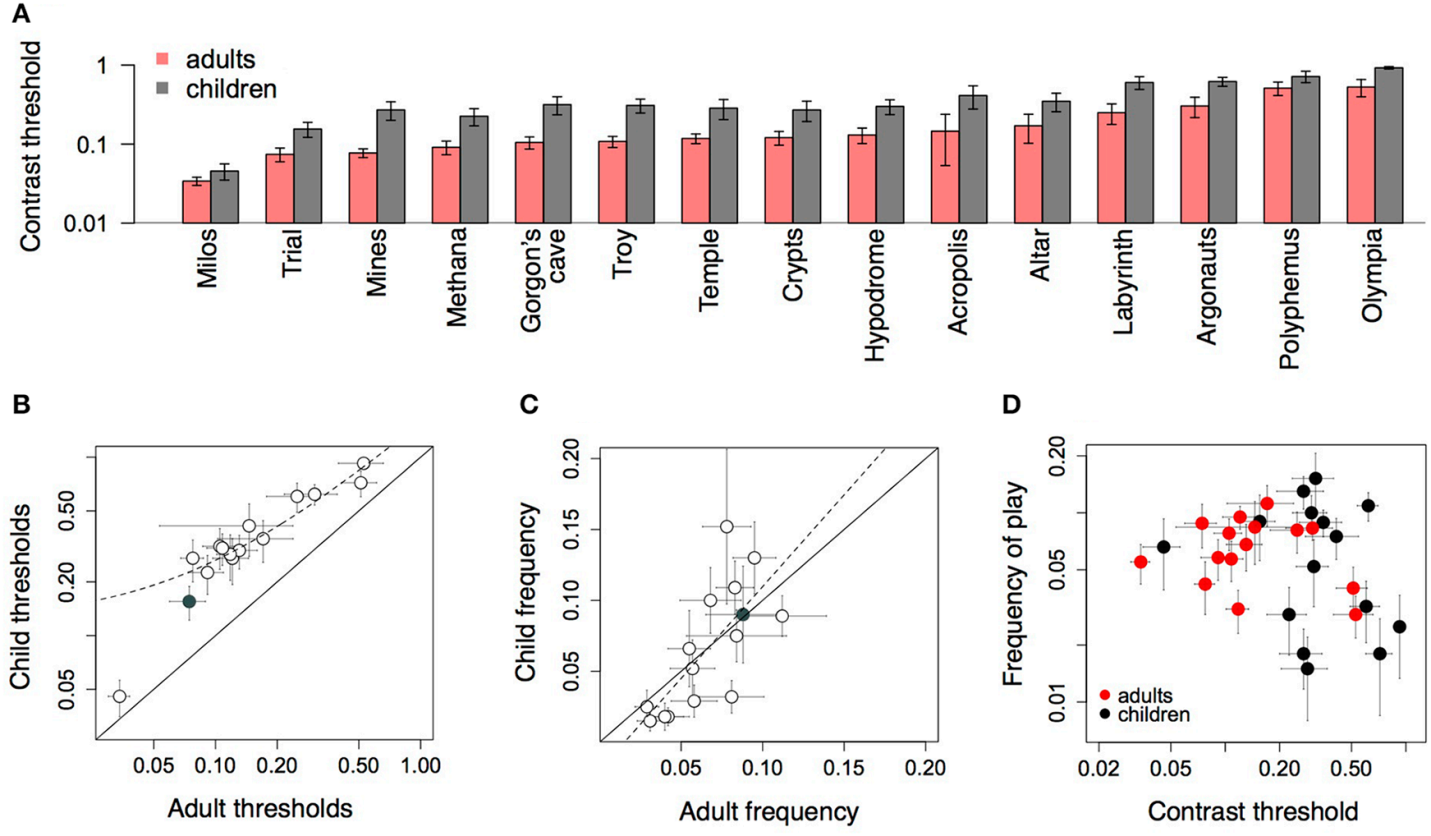
**(A)** Average performance of children and adults on each level of the game. **(B)** Children's thresholds on each level of the game plotted against adult thresholds for that level. Filled symbol shows the level Trial. **(C)** Proportion (of all levels) that each level was played across all training sessions, children vs. adults. Filled symbol shows Trial. **(D)** Average frequency that each level was played against average threshold for that level.

Figure 8A shows average performance on each of the fifteen levels of the game for children and adults. Thresholds were higher for children than adults in all levels, and varied consistently across levels for both groups. These data illustrate the variation in difficulty across levels, which arose from the dynamics of the moving objects on the screen. The level Trial was always played during each session, after which subjects played a subset of the other levels, the choice of which varied across subjects. Figure 8B shows that children's thresholds across all levels were uniformly larger than adult's thresholds, and correlated across levels [*r* = 0.95, *t*_(13)_ = 10.99, *p* < 0.0001]. On average across all levels, children's thresholds were twice adult thresholds [mean = 0.39 vs. 0.18; *t*_(24.152)_ = 2.89, *p* = 0.009].

Figure 8C shows the preference for different levels by adults and children. Each symbol shows the average proportion that a given level was played, of all levels played across all sessions. With fifteen levels in the game, if subjects were choosing all levels equally, the frequency for each level would be approximately 0.07 (i.e., 1/15). However, certain levels were more popular than other levels, and this preference was consistent across adults and children [*r* = 0.72, *t*_(13)_ = 3.71, *p* < 0.002]. The gray symbol shows the level Trial, which was obligatory on each run, and which was played equally often by both groups.

Was level preference predicted by level difficulty? Figure 8D shows the average proportion that each level was played (across all levels and sessions) against the average contrast threshold on that level, separately for adults and children. The frequency that each level was played was uncorrelated with difficulty as measured by the average threshold, both for adults [*r* = −0.32, *t*_(13)_ = −1.22, *p* = 0.24], and children [*r* = −0.27, *t*_(13)_ = −1.05, *p* = 0.31].

## 5. Discussion

Our aim was to create an engaging video game based on psychophysical tasks that achieve the largest acuity improvements in amblyopia. Contrast-based tasks (i.e., tasks in which the dependent measure is target contrast), have been linked to the largest benefits for visual acuity in amblyopia (Levi and Li, 2009; Astle et al., 2011; Levi, 2012). Therefore, targets that varied adaptively in contrast were a key aspect of the game. We tested both adults and children, who played the game for multiple sessions. The game provided reliable estimates of contrast thresholds for the adults, whose thresholds decreased with game play; thresholds were much more variable and did not change significantly after training for children. The absence of improvement in contrast thresholds of children may be related to a number of other factors discussed below. Threshold improvements notwithstanding, there was a significant improvement in logMAR acuity for both groups after training. For four subjects with mild amblyopia (initial acuities ranging from 0.14 to 0.20), the difference in acuity between the eyes decreased to less than 0.20 logMAR such that they could no longer be classified as amblyopic after training. The improvements in acuity were specific to the trained eye, and were retained when measured several weeks later for a subset of individuals who returned for a second post-test.

There are now a number of studies suggesting that playing of video games, whether off-the-shelf or customized, can improve visual acuity in amblyopia (Li et al., 2011, 2013; To et al., 2011; Hess et al., 2012; Jeon et al., 2012; Knox et al., 2012; Herbison et al., 2013). Across these studies, an improvement of approximately 1–2 lines (0.10–0.20 logMAR) was obtained after 5–40 h of training. We obtained an average logMAR improvement of 1.3 lines (range 0–3.6 lines; across both groups, and including the improvements obtained after extended practice), after an average of 11 h of training distributed over multiple sessions. An improvement in logMAR acuity of 1.3 lines within this duration compares favorably with the above reports, but it is not clear that further training would have produced more improvement. We consider below factors that may constrain improvement in game-like settings, the mechanisms of improvement in such settings, and the caveats of this study.

### 5.1. Amount of practice

An improvement of 1.5–2 lines in logMAR acuity emerges as the standard effect size from a number of studies on perceptual learning in amblyopia (see Levi and Li, 2009; Levi, 2012, for reviews). This effect size is fairly stable despite the considerable variety of tasks and practice durations used. Prolonged practice does confer additional benefits on the trained task in certain cases, (e.g., Li et al., 2007, 2008), but reports of complete resolution of amblyopia (based on the criterion of equivalent visual acuity in both eyes), are confined to cases of mild amblyopia (e.g., Li et al., 2011, and the present study). In the present case, the total number of hours played was positively correlated with logMAR improvement in children but not adults (Figure 7), and visual acuity improved slightly after additional practice only for children and not adults (Figure 6). This difference between children and adults may have arisen due to a longer initial period of procedural learning in children, due to differences in the amount of time played per session (i.e., the distribution of total practice across sessions), or simply due to differences in the maturity of sensory, cognitive and motor skills of the two groups. The maximum duration of game play here was 18.3 h, less than the 50–100 h reported to produce asymptotic performance in amblyopes on a positional discrimination task (Li et al., 2007, 2008). However, the relationship between total hours played and improvement in logMAR acuity was independent of performance on the game, which varied considerably and did not improve for children, but was more reliable, and improved in adults. In Li et al. (2007) as well, asymptotic performance on the trained task after an extended amount of practice was not accompanied by full resolution of the acuity difference between eyes. Hess et al. (2012) also have reported the absence of a correlation between total hours of play on a dichoptic video game and outcome measures including improvement on the game, improvement in logMAR acuity, and stereoacuity. This pattern of results suggests that for severe amblyopia, there may be a ceiling on the functional benefits of practice-based approaches that currently are being tested, and that the sheer amount of practice whilst beneficial for task performance *per se*, may only go so far toward improving visual acuity.

### 5.2. Task- and stimulus-related factors

#### 5.2.1. Pursuit of low contrast targets in a video game

Pursuit of moving objects mimicked the engaging aspects of action video games that are thought to activate the reward mechanisms of learning (Rokem and Silver, 2010; Baroncelli et al., 2011; Levi, 2012). First person-shooter games such as Medal of Honor most frequently linked to improved visual function in amblyopia (e.g., Li et al., 2011), involve rapid responses to salient targets. Psychophysical tasks on the other hand, call for sustained focus on a single attribute of a stimulus, which although not as stimulating, may evoke types of learning that are absent or diffuse in video games. Contrast-based laboratory tasks that thus far have produced improvement in acuity in amblyopia, have required discrimination of foveated, static targets in clearly defined spatial or temporal intervals, that is, in stimulus conditions optimized for producing low thresholds (Polat et al., 2004; Zhou et al., 2006; Chen et al., 2008; Huang et al., 2008; Astle et al., 2011). Insofar as dynamic target pursuit remains an objective of a game, threshold tasks (and especially contrast-based tasks) are not easily adapted to video games because pursuit of targets is difficult or impossible near threshold. Furthermore, smooth pursuit eye movements in strabismus are abnormal, and biased toward certain parts of the visual field and directions of motion (Schor and Levi, 1980; Tychsen and Lisberger, 1986; Demer and von Noorden, 1988; Lions et al., 2013). This asymmetry in eye movements could affect performance on a large proportion of targets presented in these conditions. In other words, certain characteristics of videogames that are optimal for learning may be incompatible with those of psychophysical tasks, curtailing the overall benefits when both methods are combined. Note however, that as far as video games go, it is not crucial that the game be an action video game, or that contrast be manipulated. Improved visual function was shown after practice of a non-action video game (SIMS), involving no manipulation of contrast (Li et al., 2011).

#### 5.2.2. Target size and other attributes

All stimulus attributes except contrast (e.g., size, speed), were above threshold and held constant across all sessions. This was done intentionally to isolate contrast as the training variable, and to minimize uncertainty associated with the other variables. However, there is evidence that near-threshold stimuli generate larger improvements than stimuli that are above threshold (Zhou et al., 2006; Huang et al., 2008). Larger improvement may have resulted here if stimulus size for instance, had been set exactly to, rather than above the acuity limit of subjects' amblyopic eye, and if this size were adjusted at the start of each session (and not just the first session).

#### 5.2.3. Monocular vs. dichoptic training

Some studies suggest that interocular suppression (i.e., inhibition of the amblyopic eye by the fellow eye), plays a large role in the acuity deficit in amblyopia, and that treatments targeted at reducing suppression may be more successful in improving the acuity of the amblyopic eye (To et al., 2011; Hess et al., 2012; Li et al., 2013). To re-establish the balance between the two eyes, these studies have used dichoptic training methods, in which visual input to the two eyes is separated, and the contribution of inputs is recalibrated with training. Using a dichoptic version of the popular game Tetris, the above studies have shown significant recovery of stereo function and improvements in visual acuity, which in certain instances exceed the improvements found with monocular methods within an equivalent duration of practice (e.g., Li et al., 2013, N.B. Due to the asymmetric crossover design used in this study, an enhancement of the dichoptic effect from prior monocular training cannot be ruled out). Given evidence for the recovery of stereo function and acuity after monocular video game play (including full recovery for a subset of mild amblyopes, e.g., Li et al., 2011), it appears that techniques aimed at reducing suppression may be sufficient, but are not necessary to improve visual function in amblyopia. The relative advantages of these two approaches remain an area of investigation.

### 5.3. Game design

What are the ingredients for a compelling video game? Features such as the stopping rule, for instance, may influence subjects' engagement over multiple sessions, or within a single session. These features are especially relevant for younger age groups less motivated by the functional benefits of game playing.

#### 5.3.1. Stopping rule

Subjects played out each 90 s level regardless of whether stimulus contrast was near threshold, and then proceeded to the next level of their choice. Achievement was based on the total number of coins earned within the 90 s period, rather than on reaching a contrast-defined target. This scenario was created to give subjects the best chance at achieving a low threshold within the specified period. On the other hand, a performance-limited stopping rule rather than a time-limited stopping rule may have better guided subjects toward achieving lower thresholds. For instance, one rule might require the player to remain at a particular low contrast for a fixed duration, or to achieve a particular target contrast before progressing to the next level. Indeed, in many popular commercial video games, players must achieve well-specified targets or else they must re-play that particular level.

#### 5.3.2. Feedback

Two sources of feedback were provided to subjects through a coin score and a contrast sensitivity score. Dual feedback may have been less effective than a single score based entirely on performance and more closely linked to the type of stopping rule described above. Various other mechanisms were included to boost subjects' interest in the game, including bonuses, auditory feedback and graphs at the end of each level showing contrast sensitivity over the 90 s period. Based on subjects' comments, we suspect that this some of this feedback was only partially effective, and not always meaningful. The link between bonuses and visual performance was sometimes not clear, and the bonuses may have distracted subjects from the targets.

#### 5.3.3. Treatment of contrast

In a time-limited game with contrast changing adaptively, there were periods when the stimuli were not visible on the screen. During such periods, we observed that subjects tend to pause or to move the mouse randomly across the screen, resembling guessing in standard 2AFC tasks. The challenge lies in minimizing the duration of such guessing periods, which reduce engagement in the game, whilst keeping stimuli at near-threshold contrast. This might be achieved through algorithms that smooth the window over which contrast changes are calculated, and by setting an artificial floor for each session that does not allow contrast to decrease beyond a certain point.

#### 5.3.4. Progression through levels

Each level in the game was a variation of target-distactor configuration and motion. Certain levels were more difficult and/or compelling than others, but were not ordered by difficulty. Subjects were free to choose which levels they played during a session, provided they had completed one run on Trial. Access to all levels was intended to keep subjects interested in the game, but guided or forced progression through levels of increasing difficulty may have created a larger sense of achievement in subjects.

### 5.4. Age, motivation, and attention of participants

Higher contrast thresholds for children than adults have elsewhere been attributed to the immaturity of the sensory system rather than non-visual, attentional factors (Liu et al., 2014). Here however, a number of factors could have contributed to childrens' larger variability in thresholds across sessions. The adult subjects were motivated by the visual benefits of the game and played the game regularly and for longer periods than the children (Figure 7). Keeping the children on task was more challenging. Several parents reported difficulty in motivating their children to play the game after the initial week. As with patching, even video games may carry issues of compliance when prescribed for children. Enhanced game design could address this issue to increase the attractiveness of the game, for instance by varying some other stimulus attribute than contrast, adding narrative elements, and changing the features described above. In the present case although motivational issues may have affected performance on the task, improvement in acuity did not differ between adults and children. Furthermore, although children's thresholds were generally higher than adults', they were strongly correlated with adults' thresholds across the different levels (Figure 8), suggesting that the variability in performance across levels was not due only to differences in motivation or skill. Children also tended to prefer the same levels as adults, suggesting that certain aspects of the game appealed to both age groups equally.

### 5.5. Mechanism of improvement and constraints on plasticity

The mechanisms of learning of basic sensory tasks and of video games continue to be investigated. Learning may reside in plasticity of low-level representations (Jehee et al., 2012), decoding or decisional rules (Law and Gold, 2008; Gold and Ding, 2013), attentional sharpening (Otto et al., 2010), or in some combination of these. Perceptual learning has also been attributed to increased sampling efficiency (Gold et al., 1999), reduced internal noise (Lu et al., 2006), or a combination of both (Dosher and Lu, 1998; Lu and Dosher, 2009). Stimulus specific learning, a characteristic of perceptual learning in the normal visual system, was until recently interpreted as evidence for plasticity of sensory representations in primary cortices (see Karni and Bertini, 1997, for an earlier review). It is now clear that stimulus specificity depends on a number of factors including task difficulty (Wang et al., 2013), the axis of generalization (Webb et al., 2007), and attributes of the training regimen (Xiao et al., 2008; Zhang et al., 2010; Hussain et al., 2012a; Hung and Seitz, 2014). Therefore, stimulus specificity (or generalization) in itself cannot isolate the neural mechanism of learning. The broader-than-normal generalization of learning found in amblyopia on contrast sensitivity tasks (Huang et al., 2008; Astle et al., 2010) and the generalization of improvements from the trained tasks to logMAR acuity could reflect higher order learning, but also may reflect the greater capacity for improvement in low-level representations in developmentally impaired visual systems. Overall, the exact mechanisms of improvement after practice of sensory tasks are not yet clear, but the scope for improvement does appear larger in the amblyopic- than in the normal visual system. Improvements in visual function through perceptual learning can be enhanced through pharmacological and environmental interventions (e.g., fluoxetine, dark exposure) that relax the constraints on neural plasticity (Baroncelli et al., 2011; Montey and Quinlan, 2011). Due to the limited practicality of these interventions, other methods of enhancing the functional benefits from perceptual learning remain an area for future work.

### 5.6. Caveats

We tested the game on a small number of subjects (10 adults and 10 children), and found a positive effect on visual acuity after playing the game for 12 or more sessions. We compared improvements in acuity between the amblyopic and the fellow eye to rule out test-retest effects, and learning on letter-based measures of visual acuity. A more comprehensive study would have included a no-training group, a no-training group that was patched for a similar duration each day as the target group, and groups that played variations of the game to isolate its relevant components. All subjects in this study played the game at home, outside the supervision of the experimenters. Children may or may not have been supervised by their parents. In the above respects, the improvements in visual acuity cannot unequivocally be attributed to game play or to sensory plasticity. We also note a number of recent studies that call into question whether perceptual and cognitive benefits truly arise from practice on video games (Boot et al., 2011; Oei and Patterson, 2014; van Ravenzwaaij et al., 2014). Larger-scale studies, using randomized controlled trial methodology are needed to establish whether video game playing improves perceptual or cognitive skills in the normal population more generally, and in clinical populations specifically.

## 6. Conclusions

This study was designed to investigate the merits of combining psychophysical methods with video games for the purpose of treating amblyopia. We found a modest improvement in logMAR acuity of the amblyopic eye after subjects played a video game in which the contrast of targets changed adaptively over time. Future work will address more effective ways of combining the above methods to enhance the total amount of improvement.

## Funding

This work was supported by the European Community's Seventh Framework Programme [FP2007-2013] under grant agreement no. 223326. Ben S. Webb was funded by a Wellcome Trust Research Career Development Fellowship. Andrew T. Astle was supported by a National Institute of Health Research (NIHR) Postdoctoral Fellowship.

### Conflict of interest statement

The authors declare that the research was conducted in the absence of any commercial or financial relationships that could be construed as a potential conflict of interest.
